# Therapeutic effects of the transplantation of VEGF overexpressing bone marrow mesenchymal stem cells in the hippocampus of murine model of Alzheimer’s disease

**DOI:** 10.3389/fnagi.2014.00030

**Published:** 2014-03-07

**Authors:** Karina O. Garcia, Felipe L. M. Ornellas, Priscila K. Matsumoto Martin, Camilla L. Patti, Luiz E. Mello, Roberto Frussa-Filho, Sang W. Han, Beatriz M. Longo

**Affiliations:** ^1^Neurofisiologia, Depto. Fisiologia, Universidade Federal de São PauloSão Paulo, Brazil; ^2^Biofísica, Universidade Federal de São PauloSão Paulo, Brazil; ^3^Farmacologia, Universidade Federal de São PauloSão Paulo, Brazil

**Keywords:** Alzheimer’s disease, memory deficits, mesenchymal stem cell, vascular endothelial growth factor, angiogenesis, amyloid plaques

## Abstract

Alzheimer’s disease (AD) is clinically characterized by progressive memory loss, behavioral and learning dysfunction and cognitive deficits, such as alterations in social interactions. The major pathological features of AD are the formation of senile plaques and neurofibrillary tangles together with neuronal and vascular damage. The double transgenic mouse model of AD (2xTg-AD) with the APPswe/PS1dE9 mutations shows characteristics that are similar to those observed in AD patients, including social memory impairment, senile plaque formation and vascular deficits. Mesenchymal stem cells (MSCs), when transplanted into the brain, produce positive effects by reducing amyloid-beta (Aβ) deposition in transgenic amyloid precursor protein (APP)/presenilins1 (PS1) mice. Vascular endothelial growth factor (VEGF), exhibits neuroprotective effects against the excitotoxicity implicated in the AD neurodegeneration. The present study investigates the effects of MSCs overexpressing VEGF in hippocampal neovascularization, cognitive dysfunction and senile plaques present in 2xTg-AD transgenic mice. MSC were transfected with vascular endothelial growth factor cloned in uP vector under control of modified CMV promoter (uP-VEGF) vector, by electroporation and expanded at the 14th passage. 2xTg-AD animals at 6, 9 and 12 months old were transplanted with MSC-VEGF or MSC. The animals were tested for behavioral tasks to access locomotion, novelty exploration, learning and memory, and their brains were analyzed by immunohistochemistry (IHC) for vascularization and Aβ plaques. MSC-VEGF treatment favored the neovascularization and diminished senile plaques in hippocampal specific layers. Consequently, the treatment was able to provide behavioral benefits and reduce cognitive deficits by recovering the innate interest to novelty and counteracting memory deficits present in these AD transgenic animals. Therefore, this study has important therapeutic implications for the vascular damage in the neurodegeneration promoted by AD.

## Introduction

The extensive deposition of amyloid-β peptide (Aβ), which forms senile plaques, in the cortex and hippocampus is one of the main pathological features of Alzheimer’s disease (AD). At present, all missense mutations that are linked to the familial form of AD are present in genes related to the metabolism of Aβ, such as the amyloid precursor protein (APP) and presenilins1 and 2 (PS1 and PS2; Bertram and Tanzi, 2004). Evidences indicate that the Aβ peptide is implicated in the vascular dysfunction present in AD. Accumulating Aβ peptide in the brain parenchyma of AD patients is responsible for reduced vascular permeability (Bergamaschini et al., 2004), leading to the loss of blood flow by capillary bed degeneration (Zekry et al., 2002). *In vitro* assays show that at high concentrations, the Aβ peptide limits the formation of new capillaries because it promotes the degeneration of endothelial cells. In *in vivo* AD studies, Aβ inhibits angiogenesis, suppresses the formation of blood vessels (Paris et al., 2004), and causes vessel disruption, which is induced by Aβ deposition in the vessel walls (Hardy and Cullen, 2006; Zhang-Nunes et al., 2006). In transgenic AD models, the animals exhibit several symptoms similar to those observed in AD patients, including Aβ accumulation in the brain parenchyma and around the blood vessels, as well as significant vascular abnormalities and cognitive and social behavioral deficits.

Mesenchymal stem cells (MSCs), which are found in the stroma of various organs, represent the most studied population of adult stem cells. Because of the ease in obtaining these cells from the bone marrow or fat tissue, MSCs are excellent candidates for cell therapies (Brazelton et al., 2000). These cells can be rapidly mobilized to ischaemic sites (Rafii et al., 2002; Majka et al., 2003), where they primarily produce microglia and endothelial cells (Zhang et al., 2002). After being transplanted into the brain, MSCs produce positive effects by reducing Aβ deposition and restoring microglial function in transgenic APP/PS1 mice (Lee et al., 2010; Kim et al., 2012a).

Angiogenic factors, particularly vascular endothelial growth factor (VEGF), which is induced during hypoxia by transcriptional factor hypoxia-inducible factor 1 (HIF-1; Leung et al., 1989), are now known to exhibit neuroprotective effects against the excitotoxicity implicated in the neurodegeneration present in AD (Greenberg and Jin, 2005). VEGF is the main regulator of vascular functions and angiogenesis, including increases in permeability, endothelial cell growth (Ferrara, 1999) and glucose transportation (Sone et al., 2000; Yeh et al., 2008). Administration of VEGF-modified MSCs prevents heart dysfunction after myocardial infarction by promoting myogenesis and angiogenesis (Gao et al., 2007) and increasing the dopaminergic differentiation in hemiparkinsonian rats (Xiong et al., 2011). In addition, combined with Ang-1, MSCs contribute to the functional recovery in cerebral ischaemia (Toyama et al., 2009). In *in vitro* experiments, VEGF binds with high affinity to Aβ, co-localizing and accumulating in conjunction with this peptide in the senile plaques of the brain parenchyma of AD patients (Yang et al., 2004). Aβ sequesters the soluble VEGF present around the senile plaques, reducing its availability for protecting cerebral vessels and neurons against the hypoperfusion that occurs in AD pathology (Wang et al., 2011). Thus, an appropriate therapeutic strategy in AD might be the supply of VEGF by local gene transfer.

Considering the similarities to clinical AD cases, the transgenic APPswe/PS1dE9 mouse model described by Jankowsky et al. (2004) represents a useful model to investigate cell-based therapies in AD. These transgenic animals have decreased vascularisation and angiogenesis rates, have accumulation of the Aβ peptide and cognitive and memory deficits. Based on these observations, the present study proposed to investigate the functional recovery of the hippocampal vasculature in a double transgenic mouse model of AD (2xTg-AD) with the APPswe/PS1dE9 mutations by transplanting bone marrow MSCs overexpressing VEGF (MSC-VEGF). Our working hypothesis was that the MSCs would promote neovascularization, which would be potentiated by high VEGF expression and contribute to the Aβ peptide clearance, consequently improving the cognitive deficits that are present in AD.

## Methods

### Subjects

2xTg-AD male congenic mice (APPswe/PS1dE9, B6.Cg-Tg(APPswe,PSEN1dE9)85Dbo/J) obtained from Jackson Laboratory (JAX® Mice and Services, Bar Harbor, Maine, USA), were bred, raised and maintained in the Center for the Development of Experimental Models in Medicine and Biology of the Universidade Federal de São Paulo. The mice were housed in polypropylene home cages (41 × 34 × 16.5 cm) in a pathogen-free facility. Animals (weighing 30–35 g) were housed under controlled temperature (22–23°C) and lighting (12 h light, 12 h dark, lights on at 6:45 a.m.) conditions. Rodent chow and water were available *ad libitum*. The animals were maintained in accordance with the National Institute of Health Guide for the Care and Use of Laboratory Animals (NIH Publication No. 8023), revised 2011. The Ethics Committee of UNIFESP approved all of the experiments under the protocol #0396/09.

### Mesenchymal stem cell preparation and transfection

Bone marrow MSCs from 6-week-old C57BL/6-Tg(ACTB-EGFP)10sb/J transgenic mice (JAX® Mice and Services, Bar Harbor, Maine, USA) were collected from femurs and tibias by flushing with culture medium (DMEM, Dulbecco’s Modified Eagle’s Medium, Gibco, San Diego, CA, USA). The cells were centrifuged and resuspended in DMEM low glucose containing inactivated 10% foetal bovine serum Gibco), 3.7 g/l HEPES (N-2-hydroxyethylpiperazine-N′-2-ethane-sulphonic acid, Sigma-Aldrich), 1% 200 mM L-glutamine 100x (Gibco) and 1% PSA (Gibco). The cell number and viability were determined by trypan blue staining (Gibco) and reached a final cell density of 5 × 10^6^ cells/ml. The cells were incubated at 37°C for 72 h, and the adherent cells, which were considered MSCs, were maintained in culture until reaching ~80% semi-confluence. Then, the MSCs were washed, incubated with trypsin-ethylenediaminetetraacetic acid (EDTA) (StemCell Technologies, Vancouver, Canada) and prepared to be frozen with a cryoprotectant solution of dimethylsulphoxide (DMSO, MP Biomedicals, Santa Ana, USA) and Fetal Bovine Serum (FBS).

For transfection, the cells were unfrozen and expanded until the 10th passage, when the transfection was performed. Human VEGF 165 cDNA from uP-VEGF (Sacramento et al., 2010) was obtained after digesting with Hind III and Xba I and inserted into the pVAX (Invitrogen) at the same sites. An insert containing the CMV promoter, VEGF and polyA sequences from this vector was excised using NruI and Pvu II and inserted into the pT2BH vector (kindly provided by Dr. Perry Hackett, University of Minnesota), which was previously treated with Eco RV. The final product was named pT2-VEGF. For transfection, 5 × 10^5^ cells were resuspended in 50 μL of SMEM, mixed with pT2-VEGF (4 μg) and pCMV-SB100X (4 μg), which expressed the sleeping beauty transposase (Mátés et al., 2009) and were electroporated applying 1,500 V/cm and 12 pulses with a 150 ms duration (BTX electroporator, MA, USA). After electroporation, the cells were seeded in 6-well plates and expanded until the 14th passage. Even though less cell passages are preferable for cell therapy, in practice more passages are required to obtain enough number of cells for transplantation. Here, we transfected MSC by electroporation, which is a very efficient method, but also increase cell unviability. Consequently, more cell passages are required to eliminate unviable cells and obtain enough number of transfected viable cells (Martin et al., 2014, in press). With appropriate MSC controls to qualify the cells, MSC cell differentiation to osteoblast and adipocyte are the best method to characterize MSC cells validating 14 passages are well for *in vivo* experimentation. VEGF expression was evaluated by ELISA (BD Biotech, Franklin Lakes, USA).

### Cell transplantation

2xTg-AD animals at 6, 9 and 12 months of age (*n* = 10 per group) were anesthetized, and 1 × 10^6^ of the cells in a 5 μL volume were stereotaxically injected in the lateral ventricle with MSCs or MSC-VEGF. The cell suspension was placed in a Narishige microinjector (Narishige Scientific Instrument Laboratory, Tokyo, Japan) guided with the stereotaxic apparatus. The following coordinates from the atlas by Paxinos and Franklin (2004) were used: −0.34 mm posterior to bregma, −0.9 mm lateral to the midline and 2.3 mm ventral to the skull surface. The age-matched wild type-saline (WT-SAL) and 2xTg-AD groups (*n* = 10 per group) were injected with saline at the same coordinates and volume (Alzheimer’s disease-saline (AD-SAL)).

### Open-field evaluation (OF)

Forty days after transplantation, mice were tested in the open-field (OF) task to evaluate general locomotor activity. The OF apparatus consisted of a circular wooden arena 40 cm in diameter, bounded by 50 cm high wall, with an open top and the floor divided into 19 segments by black painted lines on the wooden floor. The mice were exposed to the OF arena to quantify their basal general activity for 5 min. Faecal pellets were removed, and the apparatus was cleaned with 5% ethanol after every subject. During the session, the observer was unaware of the experimental design. The parameters assessed for the present studies were the total locomotion frequency of the squares/segments traversed. All of the behavioral tests were conducted under standard room lighting.

### Social recognition test (SR)

**Figure 1:**
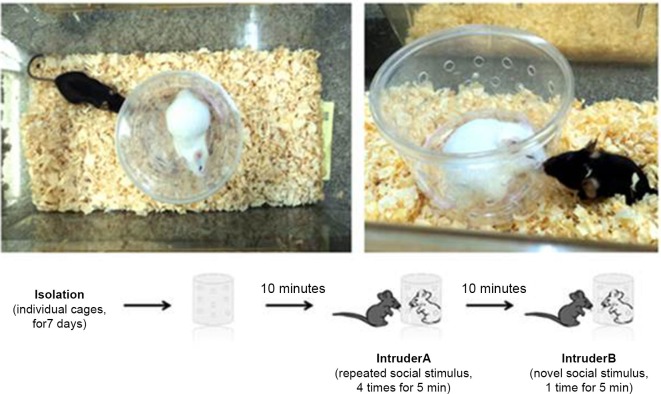
**Social recognition test**. Each session consisted of five trials of 5 min each separated by 10 min intervals. In subsequent trials, the subject mouse was exposed to the same intruder. In the last trial, a new intruder (second intruder) was placed in the same acrylic chamber (which was properly cleaned to remove the odors of the previous intruder), and the sniffing time was quantified again.

The mice were tested in the social recognition test (SR) as described elsewhere (Choleris et al., 2003; Prado et al., 2006) to assess their social recognition memory and novelty reaction. Seven days before the SR test, the animals were kept in individual cages to establish territorial dominance. Six-week-old Swiss male mice were used as intruders. Before the first trial, an empty chamber was placed in the test cage with the subject mouse to allow spontaneous exploration (Figure 1). During an “initial encounter”, an intruder was placed inside a transparent acrylic chamber with several orifices on the walls. The sessions consisted of five trials of 5 min each, separated by 10 min intervals. In the subsequent four trials, the subject mouse was exposed to the same intruder. In the last trial (5th), a new intruder (2nd intruder) was placed in the same acrylic chamber (which was properly cleaned to remove the odor of the previous intruder), and the time spent sniffing was quantified again (Figure 1). The time spent sniffing in the social interactions was scored with a stopwatch by an observer blinded to the phenotype or treatment. The duration of investigation by the host mouse, consisting of sniffing the intruder through the orifices, was summed over the course of the trial and was used as a measure of social recognition. A reduction in the time spent sniffing between the 1st and 4th trials indicated social recognition. An increase in the time spent sniffing in the 5th trial compared to the 4th trial indicated reaction to the novelty (Prado et al., 2006).

### Plus-maze discriminative avoidance task (PM-DAT)

As described elsewhere (Fernandes et al., 2013), the apparatus employed in the plus-maze discriminative avoidance task (PM-DAT) is a modified elevated plus-maze made of wood. The apparatus has two enclosed arms with sidewalls and no top (28.5 × 7 × 18.5 cm). The enclosed arms are opposite to two open arms (28.5 × 7 cm). A non-illuminated, 100 W lamp and a hair dryer were placed over the center of one of the enclosed arms (aversive enclosed arm). In the training session, each mouse was placed at the center of the apparatus, and during a 10 min period, the aversive stimuli were administered every time the animal entered the enclosed arm containing the lamp and the hair dryer and was continued until the animal left the arm. The aversive stimuli consisted of both the illumination of the 100 W light and cold air blow produced by the hair dryer. In the test session, which was performed in the same room 24 h after the training, mice were again placed in the center of the apparatus and were observed for 3 min; however, the mice did not receive the aversive stimuli when they entered the aversive enclosed arm even though the non-illuminated lamp and the hair dryer were still placed on the middle of this arm to help distinguish between the aversive and non-aversive arms. In all experiments, the animals were observed in a blind manner, and the apparatus was cleaned with a 5% alcohol solution after each behavioral session. The percentage of time spent in the aversive enclosed arm (time spent in aversive enclosed arm/time spent in both enclosed arms × 100) was calculated. Learning and memory were evaluated by the percentage of time spent in the aversive enclosed arm during training and testing, respectively. All the measures taken during the PM-DAT were obtained manually.

### Immunohistochemistry

At the end of the behavioral tests, the animals were deeply anesthetized and perfused through the heart with 50 mL of phosphate-buffered saline (PBS) followed by 200 mL of 4% paraformaldehyde at 4°C. Coronal brain cryostat sections (40 μm thick) were made between bregma −1.34 and bregma −2.80 mm, according to the stereotaxic coordinates of the mouse brain atlas (Paxinos and Franklin, 2004). Sections of the dorsal hippocampus were selected (16 per animal) to quantify the senile plaques, blood vessels and astrocytic and microglial cells by immunohistochemistry using human Aβ (6E10 antibody), CD31, Glial Fibrillary Acidic Protein (GFAP) and Iba-1 markers, respectively. Free-floating sections were washed in PBS and incubated separately overnight with the following primary antibodies, which were all diluted in PBS (except CD31, which required permeabilization with Triton-X): mouse anti-Aβ6E10 (1:300, Covance, San Diego, USA), mouse anti-CD31 (1:50, Pharmingen, San Jose, USA), mouse anti-GFAP (1:300, Dako, Glostrup Denmark) and mouse anti-Iba1 (1:300, Wako Chemicals, Richmond, USA). After incubation, the sections were washed in PBS and incubated in the ABC kit solutions (Vectashield, Vector, Burlingame, CA, EUA) for 1.5 h. The sections were stained with diaminobenzidine (DAB, Sigma-Aldrich Corporation, St. Louis, EUA) and mounted on slides and sealed with coverslips.

For the immunofluorescence, after being incubated with the primary antibody, the sections were incubated with a secondary antibody conjugated to the Alexa Fluor 546 fluorophore (1:600, Molecular Probes, Life Technologies, Grand Island, USA) for 1 h. For double-labeling, the sections were also incubated with anti-GFP antibody conjugated to Alexa Fluor 488 (1:600, Molecular Probes) for 1 h. The sections were mounted on slides and sealed with coverslips using the mounting medium with 4′,6-Diamidino-2-Phenylindole (DAPI) (Vectashield) to stain the nuclei.

### Immunohistochemical analysis

All of the slides were examined using a light microscope (Nikon 80i), and the images were captured and digitized using the Nikon ACT-1 v.2 system and analyzed with the Image J software. The quantification of the immunohistochemical analysis for Aβ plaques, astrocytes and microglial cells was performed by counting with the Image J software. Four dorsal hippocampal slices per animal (4 slices for each marker, *n* = 5/per group) and an average of eight non-overlapping fields per slice, totalling 32 fields per hippocampus for each animal were analysed at 40x magnification. In each section, nuclear profiles of GFAP and Iba1 cells and Aβ plaques nuclei were counted by an observer blind to the experimental condition. The Paxinos atlas was used to identify and delineate the analyzed regions (Paxinos and Franklin, 2004). For the quantification of microvessels, the longitudinal vessel segment between the two nodes (join points) was considered as a vessel unit. Vessels were counted in eight acquired non-overlapping images per slice section of each animal at 40x magnification, and four hippocampal slices per animal were analyzed. The total number of vessels per hippocampal slice was considered to calculate the mean for each animal and for the group. All quantifications were performed by an observer blinded to the experimental condition of each animal.

To assess the localization and differentiation of the green fluorescence protein (GFP)-positive MSCs, immunofluorescence assays using double-labeling with GFP and the appropriate above mentioned antibodies were performed. All of the images were captured and digitized using the Nikon ACT-1 v.2 system fluorescence images) or the Spinning Disc System Leica TCS SP5 (confocal images).

### Statistical analysis

The data were analyzed using a one- or two-way and repeated analysis of variance (ANOVA) followed by the Duncan, Tukey or Bonferroni tests when necessary. A probability of *P* < 0.05 was considered significant in all comparisons.

## Results

### Vascular endothelial growth factor (VEGF) production by Mesenchymal stem cell (MSC)-Vascular endothelial growth factor (VEGF) cells

The MSC-VEGF cells produced more than 2,000 pg/ml/1 × 10^6^ cells in the medium 4 days after transfection, but the production decayed to 424 ± 46 pg/ml/1 × 10^6^ cells on 10th day and this level had been maintained thereafter. Therefore, this is a reliable demonstration of stable genetic modification and of correct functioning of gene expression.

### Mesenchymal stem cell (MSC)-Vascular endothelial growth factor (VEGF) transplantation in the double transgenic mouse model of Alzheimer’s disease (2xTg-AD) mice at 6 and 9 months of age was able to recover social recognition memory and the innate interest in novelty

**Figure 2:**
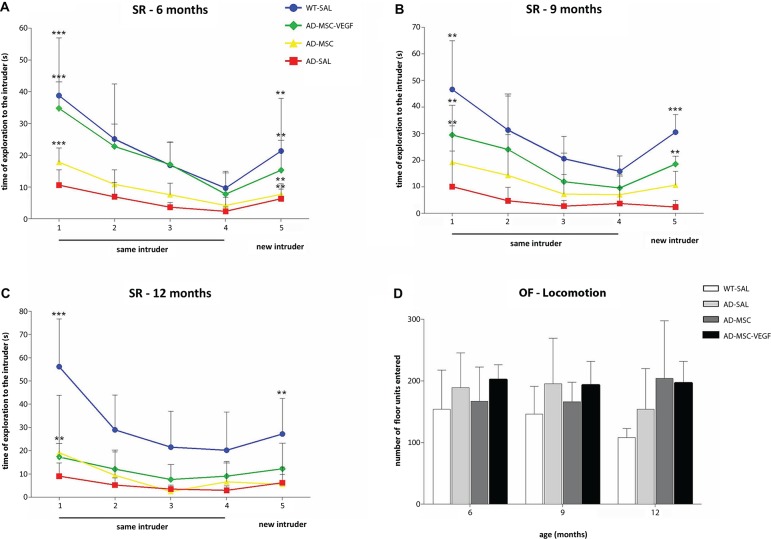
**Social recognition memory and locomotor activity evaluation**. The social recognition test (SR) in the wild type (WT-SAL) and 2xTg-AD animals (AD-SAL; AD-MSC and AD-MSC-VEGF), as evaluated by the sniffing duration (seconds) to the same intruder (session 1–4) and to a different intruder (5th session) at 6 months **(A)**, 9 months **(B)** and 12 months of age **(C)**. Repeated measures ANOVA followed by *post hoc* Bonferroni’s test (mean ± standard error, * *P* < 0.05; ** *P* < 0.01; *** *P* < 0.001, *n* = 10 per group). Differences between the first four trials are indicated in the trial number 1, and differences between the 4th and 5th trials are indicated in trial number 5. Color lines and point markers connect the mean values for the groups in each session (SAL-WT in blue; AD-MSC-VEGF in green; AD-MSC in yellow; AD-SAL in red). In **(D)**, the general locomotor activity of the WT and AD mice groups in the OF test for each age (6, 9 and 12 months), as evaluated by the total locomotion frequency of the quadrants traversed. There were no statistical differences between groups.

The ANOVA with repeated measurements for sniffing durations with treatment as a between-subject and time as a within-subject revealed that 6-month-old mice, both the control (WT-SAL) and AD animals (AD-SAL, AD-MSC, AD-MSC-VEGF), recognized the same intruder (e.g., loss of interest shown by decreased sniffing time over the four trials—a reduction in the frequency of exploratory behavior) and showed elevated sniffing times when the intruder mouse was changed in the 5th trial; this behavior was indicative of intense exploratory behavior and innate interest (repeated measures ANOVA followed by the Bonferroni multiple comparisons test; *P* < 0.0001) (Figure 2A). The WT-SAL mice and AD-MSC-VEGF-transplanted mice showed similar exploration times and interest levels in all five trials, and both groups differed from the AD-SAL and AD-MSC mice in all trials (*P* < 0.001).

At 9 months, the sniffing duration of the WT-SAL, AD-MSC and AD-MSC-VEGF animals decreased throughout the four initial trials, which was indicative of intruder recognition (repeated measures ANOVA followed by the Bonferroni multiple comparisons test; *P* < 0.0001) (Figure 2B). The AD-MSC-VEGF mice showed the same pattern of exploration time as the WT-SAL mice, and both groups maintained interest in exploring the new intruder, although the exploration time of the AD-MSC-VEGF mice was shorter compared with that of the WT-SAL mice (*P* < 0.0001).

At 12-months-old, the WT-SAL, AD-MSC and AD-MSC-VEGF mice presented a descending sniffing curve of exploration of the same intruder along the four trials (repeated measures ANOVA followed by the Bonferroni multiple comparisons test; *P* < 0.0001). AD-SAL mice did not show differences between trials at 12 (and at 9) months indicating reduction of exploration. WT-SAL also showed reduction in exploration time when comparing from 6 to 12 months, as shown by a less accentuated curve at 12 months. However, the curve for the WT-SAL mice was greater than that of the AD treated mice (*P* < 0.0001). Moreover, the AD animals (treated or untreated) lost interest in exploring the new intruder (from 4th to 5th trial), which was maintained in the WT-SAL group (*P* < 0.001) (Figure 2C).

The WT-SAL mice and all of the AD mice showed similar rates of total locomotor activity in the open-field test (Figure 2D). There were no significant variations comparing between genotypes, ages or age-treatment groups.

### Mesenchymal stem cell (MSC) and Mesenchymal stem cell (MSC)-Vascular endothelial growth factor (VEGF) transplantation distinctly modulated memory acquisition and retention in the double transgenic mouse model of Alzheimer’s disease (2xTg-AD) mice

**Figure 3:**
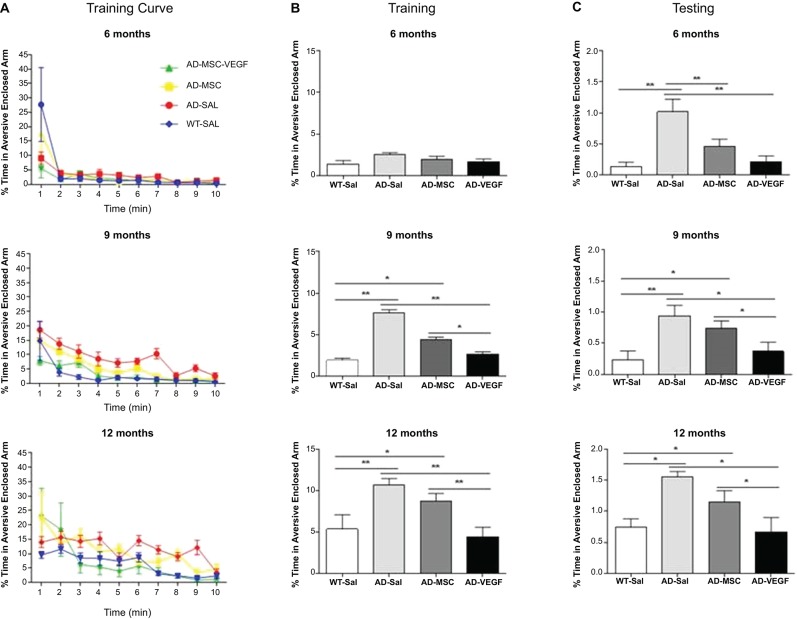
**Plus-maze discriminative avoidance task (PM-DAT) evaluation**. The PM-DAT performance of the wild type (WT-SAL) and 2xTg-AD animals (AD-SAL; AD-MSC and AD-MSC-VEGF) during the training **(A** and **B)** and testing **(C)** for the three ages. ANOVA followed by *post hoc* Duncan’s test (mean ± standard error, * *P* < 0.05; ** *P* < 0.01; *** *P* < 0.001, *n* = 10 per group). In **A**, color lines and point markers connect the mean values for the groups in minute of observation during the training (WT-SAL in blue; AD-MSC-VEGF in green; AD-MSC in yellow; AD-SAL in red).

In the training session, repeated measures ANOVA examining the percent time spent in the aversive enclosed arm parameter with treatment as a between-subject factor and time (minutes of observation) as a repeated measures factor was performed. This analysis revealed significant effects of time (*P* < 0.001), treatment (MSC × MSC-VEGF) (*P* < 0.001), as well as time × treatment interaction significant effects (*P* < 0.01) (Figure 3A). One-way ANOVA for percent time spent in the aversive arm in the session as whole showed significant effects of treatment (*P* < 0.001). In fact, the 12-month-old WT mice displayed an increased percent time in the aversive enclosed arm compared to WT mice at 6 or 9 months age. Moreover, the effect of age seemed to be more pronounced in 2xTg-AD because mice spent a progressively higher exploration of this arm as the age advanced (AD-12 m > AD-9 m > AD-6 m). In 9- or 12-month-old 2xTg-AD mice, the MSC improved the acquisition deficits of the task while the MSC-VEGF treatment recovered it to control levels. No effects were observed in 6-month-old mice (Figure 3B).

In the test session, performed 24 h after training, the ANOVA followed by Duncan’s test showed that mice at 12 months, irrespectively of genotype, spent a significant longer percent time in the aversive enclosed arm compared to their control WT groups. In addition, the treatments with MSC alone or MSC-VEFG abolished the memory impairment displayed by the 2xTg-AD mice at 6 months of age (*P* < 0.001). In 9- or 12-month-old 2xTg-AD mice, only the MSC-VEGF cells transplantation abolished the amnesia presented by these animals (Figure 3C).

### Mesenchymal stem cell (MSC)-Vascular endothelial growth factor (VEGF) transplantation promoted neovascularization in the hippocampus of the double transgenic mouse model of Alzheimer’s disease (2xTg-AD) mice

The vascularisation in the 2xTg-AD mice was evaluated at the 3 selected ages (6, 9 and 12 month-old) in the whole hippocampus by the expression of CD31 (PECAM-1) in the blood vessels.

The two-way ANOVA for this quantification revealed a significant effect for the age × treatment interaction (*P* = 0.0299). Tukey’s test showed a gradual decrease in the microvessel number from 6 to 12 months in the mice from all of the groups (*P* < 0.0001), and this difference was more robust in the AD-SAL mice at the three analyzed ages (*P* < 0.0001).

**Figure 4:**
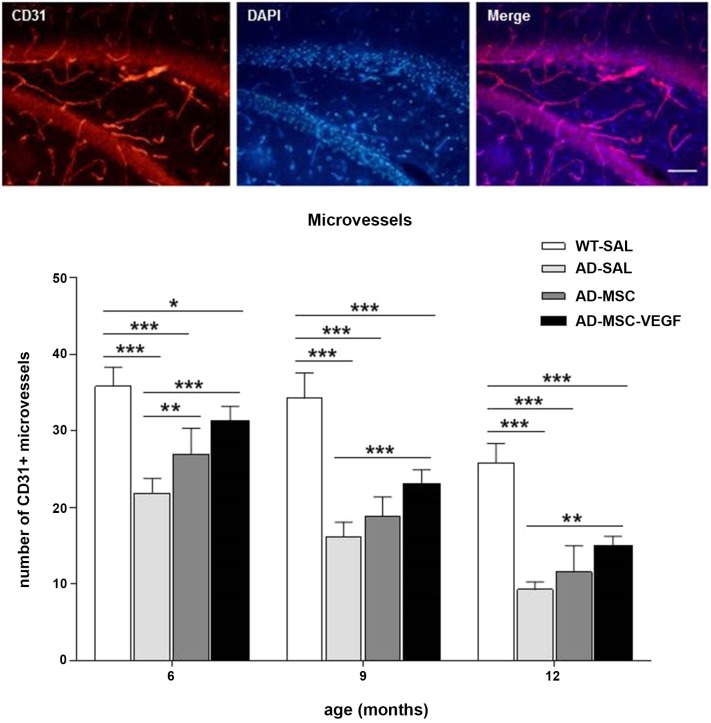
**Immunofluorescence and quantification of hippocampal vascularization**. Images above of photomicrographs of CD31 stain for vascularization in the hippocampus of AD-MSC-VEGF mouse at 9 months (scale bar 100 μm). Bellow, quantification of microvessels stained by CD31 in the hippocampus at the ages of 6, 9 and 12 months in the WT-SAL, AD-SAL, AD-MSC and AD-MSC-VEGF animals (two-way ANOVA followed by Tukey’s multiple comparisons test, mean ± standard error; * *P* < 0.05; ** *P* < 0.01; *** *P* < 0.001, *n* = 5 per group).

At the three ages, the VEGF treatment induced neovascularization compared with the AD-SAL group (*P* < 0.01) but did not result in the total recovery of the vascular density as these groups still differed from the WT-SAL group (*P* < 0.01). The AD-MSC treatment promoted the same effect at only 6 months of age (*P* < 0.01) (Figure 4).

### Mesenchymal stem cell (MSC)-Vascular endothelial growth factor (VEGF) treatments reduced the number of amyloid-beta (Aβ) plaques in the dentate gyrus compared to AD-SAL

**Figure 5:**
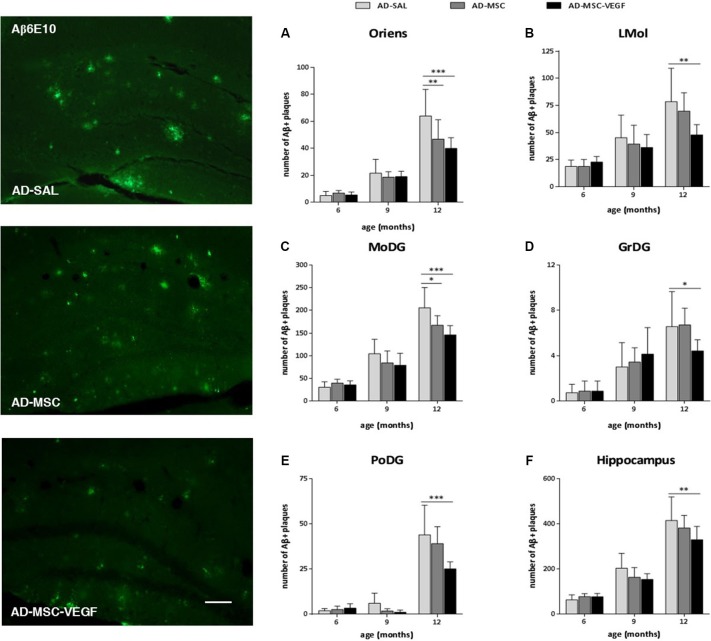
**Immunofluorescence and quantification of senile amyloid (Aβ) plaques in the hippocampal areas**. Photomicrographs of 6E10 stain for Aβ plaques in the hippocampus of 12 months mice of the three AD groups: AD-SAL, AD-MSC and AD-MCS-VEGF. Note the high concentration and large Aβ plaques in AD-SAL mouse (scale bar 50 μm). Aβ plaques stained by 6E10 were quantified in the hippocampal areas of **(A)** stratum oriens, **(B)** lacunosum molecular (LMol), **(C)** molecular (MoDG), **(D)** granular (GrDG) and **(E)** polymorphic (PoDG) layers and **(F)** in the whole hippocampus of the AD-SAL, AD-MSC and AD-MSC-VEGF transgenic animals at 6, 9 and 12 months (two-way ANOVA followed by Tukey’s multiple comparisons test, mean ± standard error; * *P* < 0.05; ** *P* < 0.01; *** *P* < 0.001, *n* = 7 per group).

To identify and quantify senile plaques (Aβ plaques) in the hippocampus, immunohistochemistry was performed using the Aβ6E10 antibody, which reacted with the 1–16 residue amino acids of the human Aβ protein (Figure 5). To evaluate the effect of the treatments in diminishing the number and the distribution of the Aβ plaques of the 2xTg-AD in the hippocampus, we quantified the senile plaques in the hippocampal stratum oriens, pyramidal, radiatum, lacunosum molecular (LMol), molecular (MoDG), granular (GrDG) and polymorphic (PoDG) layers. Because we did not detect Aβ plaques in the WT animals at any of the ages tested here, we did not include WT-SAL animals in this analysis. We compared only the AD animals of the three groups: AD-SAL, AD-MSC and AD-MSC-VEG.

The two-way ANOVA revealed an interaction effect between age × treatment, indicating that the number of Aβ plaques increased with age and was more intense in the AD-SAL animals in the stratum oriens (*P* = 0.0090), MoDG (*P* = 0.0182) and PoDG (*P* = 0.0133), whereas in the LMol and GrDG, the differences were evident only when the ages were compared (LMol, *P* < 0.0001; GrDG, *P* < 0.0001). Tukey’s test detected differences among the AD-SAL, AD-MSC and AD-MSC-VEGF mice in the stratum oriens, LMol, MoDG, GrDG and PoDG layers at 12-months-old but not at 6- and 9-months-old (*P* < 0.01) (Figures 5A–E). In the LMol, GrDG and PoDG layers, the VEGF treatment significantly decreased the number of Aβ plaques compared to the AD-SAL animals at 12 months of age (*P* < 0.01). In the stratum oriens and MoDG, this effect was detected for both treatments (MSC and MSC-VEGF) (*P* < 0.01). When considering the whole hippocampus, significant differences could still be detected in older animals (12 month-old), which indicate a decreased number of Aβ plaques in the AD-MSC-VEGF mice compared with the AD-SAL mice (*P* < 0.01) (Figure 5F). No significant difference was found for the distribution of Aβ plaques in the pyramidal and radiatum layers at any of the three ages (6, 9 or 12 month-old) among the AD-SAL, MSC and MSC-VEGF treatments.

### Mesenchymal stem cell (MSC) and Mesenchymal stem cell (MSC)-Vascular endothelial growth factor (VEGF) treatments reduced the expression of astrocytes and microglial cells in the hippocampus

The quantification of the astrocyte and microglia population in the hippocampus was performed by counting respectively GFAP, an intermediate filament protein expressed in astrocytic cells, and Iba1^+^ cells, specifically expressed in macrophages/microglia.

**Figure 6:**
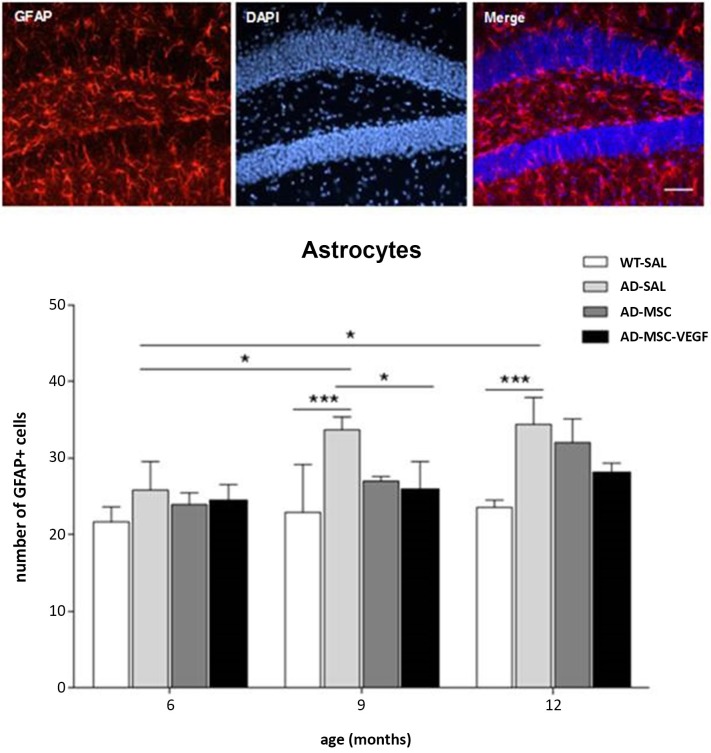
**Immunofluorescence and quantification of hippocampal astrocytic cells in the hippocampus**. Photomicrographs of GFAP stain for astrocytes in the hippocampus of AD-SAL mouse at 9 months (scale bar 100 μm). Number of GFAP^+^ cells counted in the WT, AD-SAL, AD-MSC and AD-MSC-VEGF mice at 6, 9 and 12 months (two-way ANOVA followed by Tukey’s multiple comparisons test, mean ± standard error; * *P* < 0.05; ** *P* < 0.01; *** *P* < 0.001, *n* = 5 per group).

At 6 months, no difference was detected in the GFAP^+^ cell numbers among the different experimental groups. However, the number of GFAP^+^ cells in the hippocampus of the AD-SAL mice significantly increased at 9 and 12 months compared with 6 months (two-way ANOVA, followed by Tukey’s test; *P* < 0.0001). At the ages of 9 and 12 months, the AD-SAL mice showed a higher number of GFAP cells compared with the WT-SAL mice (*P* < 0.01), and at 9 months, the MSC-VEGF treatment reduced the number of astrocytes compared with the AD-SAL treatment (*P* < 0.01), but this reduction did not reach the level observed in the WT-SAL mice (Figure 6).

**Figure 7:**
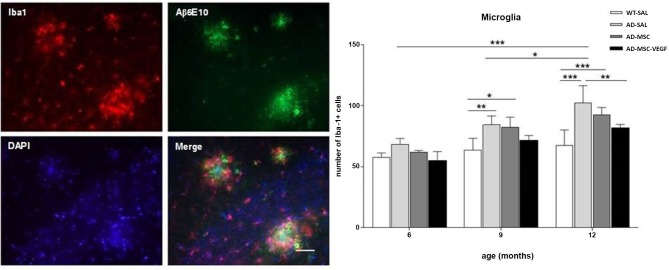
**Immunofluorescence and quantification of microglial cells in the hippocampus**. Image of double-stained senile plaques (6E10 staining in green) and Iba-1^+^ cells (in red) in the hippocampus of AD-SAL animals at 12 months. Note the co-localization of Iba-1 and 6E10 in the merged image (scale bar 200 μm). The number of Iba-1^+^ cells counted in the WT, AD-SAL, AD-MSC and MSC-VEGF mice at 6, 9 and 12 months (two-way ANOVA followed by Tukey’s multiple comparisons test, mean ± standard error; * *P* < 0.05; ** *P* < 0.01; *** *P* < 0.001, *n* = 5 per group).

The two-way ANOVA for the Iba-1^+^ cell quantification in the hippocampus revealed the interaction effects of age × treatment (*P* = 0.0328). The number of Iba-1^+^ cells increased with age (*P* < 0.0001), and the comparisons of the treatments revealed significant differences (*P* < 0.0001), except for WT-SAL vs. AD-MSC-VEGF and AD-SAL vs. AD-MSC. At 6 months, no differences were observed among the groups. At 9 and 12 months, significant differences were detected, revealing an increase in the Iba-1^+^ cells in the AD-SAL and AD-MSC groups compared with the age-matched WT-SAL groups (*P* < 0.01). At 12 months, a difference was also detected between the AD-SAL and AD-MSC-VEGF groups, indicating a decrease in the Iba-1 cells in the VEGF-treated mice (*P* < 0.001) (Figure 7).

**Figure 8:**
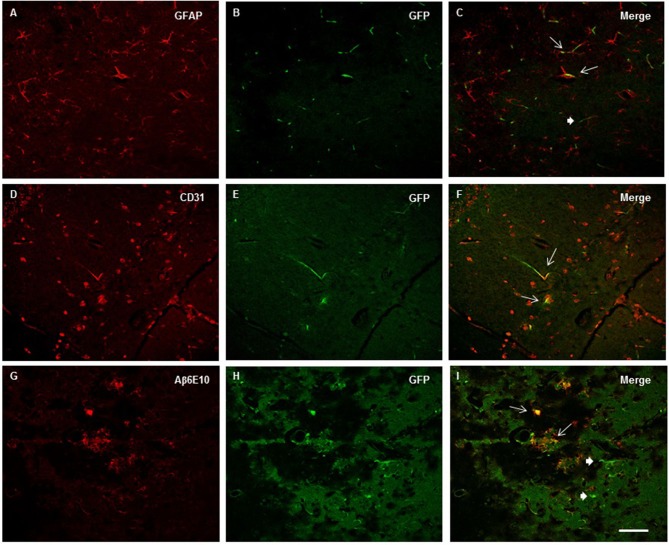
**Images from confocal microscopy reveled GFP^+^ Mesenchymal stem cells (MSC)- Vascular endothelial growth factor (VEGF) cells in the cortex (A–C) and hippocampus (D–I).** Double labeling of GFP and GFAP **(A–C)**, CD31 **(D–F)** and Aβ6E10 **(G–I)** markers were detected in mice at 6 **(A–C)**, 9 **(D–F)** and 12 months old mice **(G–I)** as indicated by thin arrows (thick for GFP^+^ cells only) in merge images **(C**, **F** and **I)**. Scale bar = 50 μm.

The qualitative analysis of GFAP and Iba-1 in 2xTg-AD animals revealed clusters of activated astrocyte and microglial cells scattered throughout the hippocampus especially near Aβ depositions (plaques). Because a function of microglial cells is clearing the Aβ protein, the location of microglia relative to the senile plaques was analyzed with immunofluorescence for double-staining of Iba-1 and Aβ6E10. The immunofluorescence analysis by confocal microscopy revealed the existence of the co-localization of clusters of Iba-1 and the Aβ plaques at the three evaluated ages (Figure 7), as well as the co-localization of GPF (MSC-VEGF) with GFAP, CD31 and Aβ6E10 markers expressed by a small amount of transplanted cells around the Aβ plaques in the cortex (Figure 8).

## Discussion

Our results indicated that the MSC-VEGF transplantation induced significant neovascularization in the hippocampus of 2xTg-AD animals, recovered the innate interest in novelty and counteracted the social and discriminative type-memories and learning deficits present in these AD transgenic animals. Additionally, the transplantation of MSCs alone or MSCs expressing VEGF reduced Aβ plaques compared to AD-SAL animals. In particular, the double transgenic APPswe/PS1dE9 (2xTg-AD) has been shown to accelerate the processes of Aβ deposition and cognitive deficits (Jankowsky et al., 2004; Garcia-Alloza et al., 2006). Using different animal models of AD, recent studies have documented the beneficial effects of MSCs from different sources to treat the basic memory deficit and formation of amyloid plaques (Lee et al., 2010; Babaei et al., 2012; Kim et al., 2012a,b).

The social recognition paradigm is a model of social memory dependent on hippocampal function (Kogan et al., 2000) that can be used in pathophysiological processes, such as ischemia and aging, which are known to interfere with these processes (Terranova et al., 1994; Prediger and Takahashi, 2005) or yet, in social memory deficits caused by a reduction in cholinergic tone (Choleris et al., 2003), also present in AD mouse models (Bories et al., 2012). In our hands, using this resident-intruder paradigm, the transplantation of MSCs transfected with VEGF vector was able to improve the social recognition memory and recover the novelty component of short-term memory impaired in 2xTg-AD animals. However, in older animals, this condition was worsened by the accentuated neurodegeneration caused by the Alzheimer’s genotype, and the robust effect of the VEGF in improving the social memory deficit in younger animals was attenuated in older animals and could not reverse the impairment of the social recognition memory at that age. Interestingly, in 2xTg-AD mice, the exploration time in each session was reduced as the age increased indicating a typical aging apathy in this AD model.

In the PM-DAT, the avoidance of the aversive enclosed arm upon testing has been validated as a measurement of retention, because amnestic manipulation decreases this effect (Patti et al., 2010). Using this animal model, we demonstrated that in the training session 2xTg-AD decreased learning levels at 9 and 12 months, as demonstrated by the increased time spent in the aversive enclosed arm. At these ages, MSC attenuated and MSC-VEGF abolished this learning deficit reaching the basal levels at the respective age-matched mice. In testing session, 2xTg-AD mice showed impaired retrieval of the memory task at the three evaluated ages in a gradually aging-dependent manner: 12 month mice showed higher magnitude deficit than 6 and 9 months. MSC-VEGF treatment was able to recover memory from deficits present in 2xTg-AD at the three ages (6, 9 and 12 months), while MSC treatment reduced memory deficits only at 6 months.

Concerning the histological findings, the 2xTg-AD animals exhibited a significant reduction in vascular density, which worsened with age. The transplantation of MSC-VEGF promoted an important neovascularization in the 2xTg-AD animals, even in older animals. This neovascularization may be primarily attributed to a paracrine signaling effect of the MSC-VEGF, as only an inexpressive number of transplanted cells was detected in the hippocampus and in the cortex. These data are in accordance with other authors that could not detect MSCs after 3 weeks post-injection (Khoo et al., 2011), or did not describe the presence or differentiation of the cells after transplantation into the brain (Lee et al., 2010; Babaei et al., 2012). We must consider that MSCs alone also secreted VEGF, although this secretion did not clearly increase the number of microvessels. However, the overexpression of VEGF by MSCs robustly affected the vascular density, which was consistent with a crucial role of VEGF in vascular function and consequently in cognitive capacity.

The results of the two memory tests indicated therapeutic effects of VEGF at the three evaluated ages. The data are consistent with that observed for neovascularization in animals treated with VEGF when compared to saline-treated 2xTg-AD. The treatment did not result in a total recovery of the vascular density as observed when compared with WT animals. These data are also consistent with a proportional decrease in memory tests observed here, suggesting a therapeutic benefits of VEGF by neovascularization.

Cerebrovascular diseases and aging in the cerebral arteries have been proposed to be connected to the pathogenesis of AD (Nicoll et al., 2004; Bories et al., 2012). These biological processes are intimately related to angiogenesis, and VEGF is the main regulator of them. In the brains of AD patients the soluble VEGF concentration is decreased because Aβ binds to VEGF forming aggregate that leads to the loss of angiogenic and neuroprotective activities (Yang et al., 2004). Therefore, provide on-site of VEGF should have high therapeutic effect, as we showed here using mesenchymal cells overexpressing VEGF that recovered the memory deficit and had high neovascularization in the hippocampus of the 2xTg-AD animals. These findings corroborate the results of Wang et al. (2011), who found that the intraperitoneal injection of VEGF enhanced hippocampal angiogenesis and decreased Aβ deposition in the brain, improving cognitive function (Wang et al., 2011).

A different approach has been proposed in a study using voluntary physical exercise in 3xTgAD model (García-Mesa et al., 2011). In this study, physical exercise improved motor performance and learning capabilities of 3xTg-AD Alzheimer-like transgenic mice. Moreover, exercise also reduced oxidative stress and showed beneficial effects on hippocampal physiology. However, although physical exercise induced positive effects on synapse strength, redox homeostasis, and general brain function, it was not able to reduce the hippocampal levels of Aβ deposition.

Fibrillar deposits of Aβ accumulated in the brains of 2xTg-AD mice as they aged. Among the animals transplanted with MSC-VEGF, a reduction in the number of Aβ plaques was evident in the hippocampus, particularly in the dentate gyrus layers, which are areas of high incidence of plaques in the 2xTg-AD mice. These results raised the question of whether the cognitive improvements were caused by reduced Aβ plaques or the increase in the hippocampal vasculature. The 2xTg-AD mice showed memory deficit and a possible explanation would be the increase of Aβ deposition. In fact, the intervention with MSC improved social and discriminative performance and decreased Aβ deposition in the hippocampus. However, not in all groups this effect was evident, and could not be explained by the presence of the cells in the brain. As suggested by other authors, the number of MSCs decreased over time when transplanted into the brain (Khoo et al., 2011), as well as into other tissues (Iso et al., 2007). Thus, another possibility was the ability of MSCs to secrete factors together with the effect of VEGF on vascularization. The animals treated with MSCs overexpressing VEGF showed better cognitive function compared to those treated only with MSCs, suggesting that neovascularization should be more relevant to the cognitive improvement in these animals, and the reduction of Aβ plaques in the hippocampus being a consequence of this neovascularization. Thus, it is imaginable that an increase in the brain vasculature could avoid the Aβ deposition, consequently improving the cognitive functions.

As has been shown in a previous study, the amount of Aβ deposition were not associated to learning and memory impairments observed in 18-month-old mice (Gruart et al., 2008). Wild-type mice that did not show Aβ deposition also presented motor, emotional, and learning deficits at this age. Accordingly, our study described a gradual impairment in social recognition memory in WT mice over the aging processes (from 6 to 12 months), WT animals also showed reduction in exploration time when comparing from 6 to 12 months. However, the curve for the WT mice was greater than that observed for AD mice. Indeed, WT animals at 12 months old showed deficit in social memory when compared to 9 and 6 months old WT mice. Moreover, even WT animals lost memory acquisition and retention learning in older ages. The 12-month-old WT mice displayed memory deficit compared to WT mice at 6 or 9 months age. Even so, the effect of age seemed to be more pronounced in AD mice. WT mice still show important differences compared to 2xTg-AD mice.

The increase in the Aβ peptides in the brain and their subsequent deposition into plaques led to an activation of the surrounding microglia and astrocytes (Cagnin et al., 2001). In fact, as an effect of genotype and aging, the AD mice showed an increased in astrocytic activity, which was reduced in the VEGF treated group at 9 months. In older transgenic animals (12 months), the MSC or MSC-VEGF treatments were able to keep the astrocytic activation levels similar to those found in the age-matched wild type animals, but did not reduce the number of astrocytes compared to the transgenic AD non-treated levels. It has been suggested that reactive astrocytes are present in large numbers in areas most affected by the disease. The GFAP-positive reactive astrocyte responses were associated with the increase of Aβ plaque deposits (Wilhelmsson et al., 2006; Kamphuis et al., 2012). Regarding the reduction of the total number of astrocytes that occurs in animals treated with MSC and MSC-VEGF, it is likely caused by the decrease in reactive astrocytes that might occur as a reduction of inflammation and number of plaques mainly due to the VEGF treatment. Older AD animals displayed large amounts of Aβ in the hippocampus, which could suggest that the astrocytic stimulation would be a mechanism of neuroprotection against Aβ and other insults.

One important role of microglia in AD that has been recently accepted is the clearance of the Aβ protein by phagocytosis (Simard et al., 2006; Lee et al., 2009, 2010). These studies documented the beneficial neuroprotective effects of microglial activation in removing β-amyloid plaques and decreasing the inflammatory responses, thus controlling the progression of AD. The decrease in the number of plaques might have been produced by phagocytosis of resident cells possibly stimulated via a paracrine effect of the transplanted cells associated with the plaques. In an elegant experiment, Simard et al. (2006) identified two distinct origins of microglia in the AD brain and showed that bone marrow-derived microglia were able to remove β-amyloid from the extracellular environment whereas the resident microglia had no effect on the presence of β-amyloid plaques. Lee et al. (2009) showed that MSC transplantation stimulated microglial activation in the brains of APP/PS1 mice that, in turn, had neuroprotective effects, modulating inflammatory responses and improving the cognitive decline associated with Aβ deposits. In accordance with these studies, herein, we observed the formation of clusters of reactive microglia that co-localized with the senile plaques. As 2xTg-AD animals aged, there was an increase in the number of microglia that was stable in wild-type animals as a function of age. The VEGF treatment was able to decrease the number of microglia to the WT levels compared with the untreated 2xTg-AD transgenic animals. The number of microglia might have decreased in the VEGF-treated mice as a consequence of the reduced number of β-amyloid plaques in the hippocampus.

### Conclusion

Our findings suggest that the overproduction of VEGF by MSCs (and possibly several other soluble factors secreted by MSCs) favored neovascularization and the clearance of Aβ protein, which ultimately recovered memory and learning deficits present in these 2xTg-AD transgenic animals. In addition to explaining the effects of MSCs in the pathophysiology of these AD transgenic animals, this study has important therapeutic implications for the vascular damage in the neurodegeneration promoted by AD.

## Author contributions

Karina O. Garcia conceived the study, carried out the laboratory experiments, analyzed the data and performed the statistical analysis; Felipe L. M. Ornellas carried out the laboratory experiments, data collection and immunohistochemistry; Priscila K. Matsumoto carried out the mesenchymal stem cell preparation and VEGF transfection; Camilla L. Patti carried out the discriminative avoidance task, the statistical analysis and critically revised the paper; Roberto Frussa-Filho (in memoriam) although helped to draft the manuscript, to interpret the results and critically revised the paper, he passed away prior to submission; Luiz E. Mello and Sang W. Han helped with the general idea of the paper; contributed with the reagents/materials/analysis tools, and critically revised the paper; Beatriz M. Longo conceived and designed the general idea of the paper, interpreted the results and wrote the paper. The work presented here was carried out in collaboration between all authors. All authors read and approved the final manuscript.

## Conflict of interest statement

The authors declare that the research was conducted in the absence of any commercial or financial relationships that could be construed as a potential conflict of interest.

## Abbreviations list

2xTg-AD: double transgenic mouse of AD
Aβ: amyloid-beta
AD: Alzheimer´s Disease
APP: amyloid precursor protein
DMEM: Dulbecco’s Modified Eagle’s Medium
GFP: green fluorescence protein
GrDG: granular layer
HIF-1: hypoxia-inducible factor 1
LMol: lacunosum molecular
MoDG: molecular layer
MSCs: mesenchymal stem cells
OF: open-field
PM-DAT: plus-maze discriminative avoidance task
PoDG: polymorphic layer
PS1: presenilins1
SR: social recognition
VEGF: vascular endothelial growth factor
